# Stroke Dysbiosis Index (SDI) in Gut Microbiome Are Associated With Brain Injury and Prognosis of Stroke

**DOI:** 10.3389/fneur.2019.00397

**Published:** 2019-04-24

**Authors:** Geng-Hong Xia, Chao You, Xu-Xuan Gao, Xiu-Li Zeng, Jia-Jia Zhu, Kai-Yu Xu, Chu-Hong Tan, Ruo-Ting Xu, Qi-Heng Wu, Hong-Wei Zhou, Yan He, Jia Yin

**Affiliations:** ^1^Department of Neurology, Nanfang Hospital, Southern Medical University, Guangzhou, China; ^2^Department of Neurology, The First People's Hospital of Zunyi, Zunyi, China; ^3^State Key Laboratory of Organ Failure Research, Division of Laboratory Medicine, Zhujiang Hospital, Southern Medical University, Guangzhou, China; ^4^Microbiome Medicine Center, Division of Laboratory Medicine, Zhujiang Hospital, Southern Medical University, Guangzhou, China

**Keywords:** stroke, brain injury, microbiota dysbiosis, fecal microbiome transplantation, outcome

## Abstract

**Background:** Significant dysbiosis occurs in the gut microbiome of stroke patients. Condensing these broad, complex changes into one index would greatly facilitate the clinical usage of gut microbiome data. Here, we formulated a gut microbiota index in patients with acute ischemic stroke based on their gut microbiota dysbiosis patterns and tested whether the index was correlated with brain injury and early outcome.

**Methods:** A total of 104 patients with acute ischemic stroke and 90 healthy individuals were recruited, and their gut microbiotas were compared and to model a Stroke Dysbiosis Index (SDI), which representing stroke-associated dysbiosis patterns overall. Another 83 patients and 70 controls were recruited for validation. The association of SDI with stroke severity (National Institutes of Health Stroke Scale [NIHSS] score) and outcome (modified Rankin scale [mRS] score: favorable, 0–2; unfavorable, >2) at discharge was also assessed. A middle cerebral artery occlusion (MCAO) model was used in human flora-associated (HFA) animals to explore the causal relationship between gut dysbiosis and stroke outcome.

**Results:** Eighteen genera were significantly different between stroke patients and healthy individuals. The SDI formula was devised based on these microbiome differences; SDI was significantly higher in stroke patients than in healthy controls. SDI alone discriminated stroke patients from controls with AUCs of 74.9% in the training cohort and 84.3% in the validation cohort. SDI was significantly and positively correlated with NIHSS score on admission and mRS score at discharge. Logistic regression analysis showed that SDI was an independent predictor of severe stroke (NIHSS ≥8) and early unfavorable outcome (mRS >2). Mice receiving fecal transplants from high-SDI patients developed severe brain injury with elevated IL-17^+^ γδ T cells in gut compared to mice receiving transplants from low-SDI patients (all *P* < 0.05).

**Conclusions:** We developed an index to measure gut microbiota dysbiosis in stroke patients; this index was significantly correlated with patients' outcome and was causally related to outcome in a mouse model of stroke. Our model facilitates the potential clinical application of gut microbiota data in stroke and adds quantitative evidence linking the gut microbiota to stroke.

## Introduction

Ischemic stroke imposes a heavy burden on society, with 24.9 million cases worldwide (1). Although intravenous thrombolysis and endovascular treatment greatly improve some patients' prognosis, the prognosis for most patients with acute ischemic stroke is still poor. Therefore, identifying potential risk factors associated with stroke prognosis is important in clinical management.

In current clinical studies, stroke patients often display significant changes in microbial diversity and bacterial counts in fecal samples independent of certain comorbidities (hypertension, age and type 2 diabetes) (2–4). In a relatively large sample size, our previous study found that, compared to asymptomatic controls, patients with acute atherosclerosis stroke showed significant dysbiosis in the gut microbiota, with increases in opportunistic pathogens and decreases in beneficial genera, and such changes were especially pronounced in severe stroke patients (3). However, clinical studies remain limited in their ability to delineate the potential link between gut microbiome and stroke and characterize the underlying mechanisms of microbiota changes in these patients. The main challenges were the large number of microbes that differed between patients and healthy individuals and the heterogeneous nature of patient clinical pathology, diets and lifestyle, all of which have major influences on the composition of the gut microbiome.

Microbiota-based models may be an alternative method to facilitate the use of data on the gut microbiota in certain diseases. Disease-related models based on gut microbial alternation patterns or microbiota-targeted biomarkers specific to certain diseases were recently suggested as powerful tools for disease risk assessment, diagnosis, and even prognosis (5–8). A microbiota-based model of inflammatory bowel disease (IBD) showed high accuracy in disease diagnosis and, furthermore, reflected disease activity and treatment efficacy (6). Stool microbiota composition in cirrhosis patients was successfully used to predict the 90-day hospitalization rate, independent of clinical predictors (8).

Interaction between gut microbiota and stroke outcome was recently reported via animal experiments (9–12). Within animals, disturbance in the gut microbiota promoted an increase in the abundance of intestinal pro-inflammatory T cells and led to exacerbation of ischemic brain lesions along with worsened stroke outcomes (11, 13). A previous study has demonstrated the correlation between the gut microbiota dysbiosis and the severity of brain injury via two distinct stroke models (13), in which gut microbiota dysbiosis and stroke severity were influenced and interacted as both cause and effect. However, there may be some concerns about the little direct information regarding the role of the patients' intestinal flora on brain injury. Indeed, the role of human flora is somewhat differed from the role of the animal flora in convenient experimental animals (14). The human flora-associated (HFA) animals, established via the fecal microbiota transplantation (FMT) technology, is a stable model for studying the ecosystem and metabolism of the human intestinal flora (14–16). Studies using HFA animals will provide much needed information on the precise role of the intestinal flora in relation to humans (14).

Here, we applied microbiota-related analysis by sequencing 16S ribosomal RNA genes to contrast the gut microbiota of 104 patients with acute ischemic stroke with those of 90 healthy participants, and we developed a Stroke Dysbiosis Index (SDI), which was established based on the profound difference in microbial taxonomic features between patients and healthy individuals. An additional 153-member group was recruited to validate this microbial model. Furthermore, associations between SDI and patients' stroke severity and early outcome were assessed. Experimental stroke model was performed in HFA mice to explore the potential causality of patients' disturbed gut microbes on stroke brain injury.

## Materials and Methods

### Ethics Statement

Raw sequence data and related metadata can be accessed at the European Nucleotide Archive (ENA) under the accession number PRJEB31562 and PRJEB31561. The study was conducted in accordance with the principles of the Declaration of Helsinki and received approval from the Ethical Committee of Southern Medical University. Written informed consent was obtained from all study subjects or their legal guardians by neurologists prior to data and sample collection.

Animals used in this experiment were conducted in accordance with the National Institutes of Health guide for the care and use of Laboratory animals (NIH Publications No. 8023, revised 1978). The protocols were approved by the Animal Ethics Committee of Nanfang hospital, Southern Medical University.

### Participants

We recruited patients with large-artery atherosclerotic ischemic stroke according to the Trial of Org 10172 in Acute Stroke Treatment [TOAST] system in this study. A 194-member cohort including 104 acute stroke patients and 90 healthy individuals was recruited from February 2014 to February 2016 for the establishment of SDI model. All of them were recruited from the Nanfang Hospital of Southern Medical University, Guangzhou, China. The patient group was recruited from the Department of Neurology and all of them were diagnosed with acute ischemic stroke according to standard clinical criteria, with supporting brain imaging (the magnetic resonance imaging or magnetic resonance angiography). Diagnosis of ischemic stroke was confirmed for all participants at admission for the index stroke. Inclusion criteria were as follows: (1) age between 18 and 80 years, (2) diagnosed with acute ischemic stroke, (3) large-artery atherosclerosis stroke subtype, (4) admission within 7 days of ischemic stroke onset, (5) collection of fecal samples within 48 h of admission. Exclusion criteria were as follows: (1) antibiotics, prebiotics, or probiotics used within 1 month before admission or after admission; (2) admission after 7 days of stroke onset; (3) death within 7 days of stroke onset; (4) a clear cause of stroke unrelated to atherosclerosis (e.g., cervical artery dissection, perivascular procedural stroke, cardioembolism stroke, or the other TOAST subtypes), (5) history of systemic disease such as cirrhosis, renal failure, gut disease (i.e., IBD, Crohn's disease and Ulcerative colitis) and malignant tumor. According to the location of stroke lesion assessed with magnetic resonance images of admission, patients were divided into two groups: (1) anterior circulation stroke group and (2) posterior circulation stroke group.

The control group was recruited from the Health Examination Center. Inclusion criteria were as follows: (1) age between 18 and 80 years, (2) willing to offer fresh fecal samples. Exclusion criteria were as follows: (1) antibiotics, prebiotics, or probiotics used within 1 month before admission; (2) history of cerebrovascular diseases; (3) history of systemic disease such as cirrhosis, renal failure, gut disease (i.e., IBD, Crohn's disease and Ulcerative colitis) and malignant tumor. All of the participants were not treated with any type of antibiotic or probiotic within 1 month before participating in our study. For all participants, fresh fecal samples were frozen at −80°C immediately after collection. The cohort study was approved by the Ethical Committee of Southern Medical University.

For validation purpose, an additional 153-member cohort including 83 patients and 70 healthy individuals was recruited for the validation of SDI model using the same selection criteria from January 2017 to December 2017. Patients were recruited from the Nanfang Hospital and Yanling Hospital of Southern Medical University. Controls were recruited from the Health Examination Center of Yanling Hospital of Southern Medical University.

### Stroke Severity and Outcome Measures

Stroke severity at baseline was assessed with the National Institutes of Health Stroke Scale (NIHSS) score by two certified neurologists. Patients were categorized according to the stroke severity into 2 groups: mild stroke, when the NIHSS score was <8; severe stroke when the NIHSS score was ≥8 (17). Functional outcome was evaluated at discharge by the modified Rankin scale (mRS) score. This observational scale provides a clinical handicap score reflecting interference with lifestyle and independent living. It ranges from 0 (no symptoms) to 6 (death). If raters judged 0–2 (*n* = 140), this was indicative of good or functional independent outcome, and if rates judged 3–6 (*n* = 47), this was indicative of poor outcome. Favorable outcome was defined as mRS 0–2. Unfavorable outcome was defined as mRS>2 (18). Demographic, clinical, and laboratory were systematically registered in a standardized format.

### Extraction, PCR, and Sequencing of Fecal Microbiota Samples

DNA extraction and Polymerase Chain Reaction Amplification of the bacterial 16S rRNA gene V4 region and sequencing were induced as our previous report (3).

### Microbiological Investigation of Fecal Samples

We used QIIME (1.9.1) for microbiota data analyses. All samples were normalized to 8,000 sequences to avoid deviation caused by the effects of different sequencing depths. The UniFrac distance was applied to analyze beta diversity (19). The principle coordinate analysis (PCoA) is a dimensionality reduction method for illustrating the relationship between samples based on a distance matrix. PCoA could be used to visualize the unsupervised grouping pattern of a complex data set such as a microbiome. Chosen information related to a microbiome can be shown as either a clear separation or a trend in PCoA by coloring samples. The linear discriminant analysis (LDA) effect size (LEfSe) was applied to determine differential taxa between groups (20). LEfSe is an algorithm for high dimensional biomarker discovery that can identify metagenomic features characterizing differences between two or more biological conditions. After coupling standard tests for statistical significance with additional tests encoding biological consistency and effect size, features that were most likely to explain the differences between the classes were determined by LEfSe analysis. The LDA threshold was set at 2. The LDA score was calculated for each of the differential features detected by LEfSe, and a higher score represented greater differences in features between the tested groups.

### Stroke Dysbiosis Index

Firstly, the command *filter_otus_from_otu_table.py–i input.biom–o output.biom –min_count_fraction 0.001* was used to filter otus abundance that lower than 0.1%. Secondly, the command s*ingle_rarefaction.py –i input.biom –o output.biom -d 4,800* was used for normalization. Thirdly, the command *group_significance.py* was used to select the differential genera (FDR-_*P* < 0.1), and 18 genera were selected. At last the formula (1) was used to calculate the SDI.

(1) $$\begin{array}{l} \text{SDI}=\{\frac{\sum_{i\;=\;7}\text{abundance}\left(\text{stroke enriched}\right)\text{i}}{7}\\ -\;\frac{\sum_{j\;=\;11}\text{abundance}\left(\text{control enriched}\right)\text{j}}{11}\}\times 100 \end{array}$$

### Animal and Housing

Six week-old male C57BL/6 mice (purchased from the Animal Experimental Center of Guangdong Province, China) were placed in autoclaved, individually ventilated cages, and kept on a 12-h light/dark circle with *ad libitum* access to food and water. Animals were randomized to treatment groups. All analyses were performed by investigators blinded to group allocation.

### Fecal Transplantation and Treatment to Human Flora-Associated (HFA) Animals

In order to acquire representative fecal material for each group described by Diao et al. (21), feces of 3 individuals of higher SDI or lower SDI were mixed in 10 mL sterile phosphate buffered saline (PBS) as described by Zeng et al. (15). HFA mice were established as the recolonization mice were challenged 0.2 mL of the supernatant from the fecal suspension by oral gavage for 2 weeks after microbiota depletion by 3 days' pulse-treated antibiotic (vancomycin 10 mg/mL, metronidazole 20 mg/mL, gentamycin 4 mg/mL, ampicillin 20 mg/mL, deployed with PBS, 0.2 mL each mouse).

### Experimental Stroke (Middle Cerebral Artery Occlusion)

The fecal recipient mice were anesthetized by annotation with 0.4 mL 1.25% tribromoethanol and subjected to 60 min' middle cerebral artery occlusion (MCAO) described as previously mentioned (22). Each group contained 10 mice. Data was excluded if mice died after surgery or without induction of brain ischaemia as quantified postmortem by histological analysis.

### Neurobehavioral Examination

Modified neurological severity score (mNSS) system was used at day 1 after MCAO to assess the neurobehavioral tests (23), with a range from 0 to 14, in which 0 represents normal and 14 represents the highest degree of neurological deficiency.

### Measurement of Infarct Volume

Infarct volume was stained by 1% 2,3,5-triphenyltetrazolium chloride solution at day 3 after MCAO. At 72 h post MCAO surgery, the perfused brains were removed and frozen immediately on powdered dry ice. The removed brain tissue was cut at 400-μm intervals into coronal cryosections 20 μm thick. The cryosections were further stained with 1% 2,3,5-triphenyltetrazolium chloride (TTC) consistent with standard protocols and fixed by formaldehyde. Finally, stained brain sections were scanned at 600 dpi. Image analysis software (MCID; Imaging Research) was used for quantifying the infarct volume after correcting the swelling. We used an edema correction formula for the infarct area as follows: (ischemic area) = (direct lesion area) – [(ipsilateral hemisphere) – (contralateral hemisphere)]. The total cerebral infarct area was calculated by integrating the measured areas of different sections in one mouse brain tissue (11).

### Cell Isolation From Spleens

Cells of spleens were prepared according to Benakis et al. (11). The spleens were isolated and placed on a premoistened 70-μm Cell strainer and homogenized with the end of a 1-ml syringe plunger. Ten milliliter erythrocyte lysis buffer (150 mM NH_4_Cl, 1mM KHCO_3_, 0.1 mM EDTA; pH 7.2) were used to wash the strainer and the eluted cells were incubated for 5 min at room temperature and washed with 40 ml PBS. Cell suspensions were centrifuged at 500 g for 7 min and subsequently stained for flow cytometric analysis.

### Cell Isolation of Intestinal Intraepithelial Lymphocytes (IELs)

Cell of the intestinal intraepithelial lymphocytes were prepared according to Benakis et al. (11). Mice were sacrificed via overdose pentobarbital (Sigma; 200 mg per kg body weight (mg/kg); i.p.). Small intestines of mice were removed and separated; peyer's patches were excised and intestines were cleaned of mesenteric fat and intestinal contents. Then we opened the intestines longitudinally, washed them with PBS, cut them into ~1-cm pieces and placed into 20 ml of HBSS with 10 mM HEPES, 4 mM EDTA, 8% FBS, 0.5 mM dithiothreitol. We washed tissue pieces three times in a shaking incubator set at 250 r.p.m. and 37°C for 20 min. After each round, tissue suspension was vortexed for 20 s and the supernatant containing the IELs was collected. The supernatants were combined and filtered first over 0.3 g of premoistened nylon wool placed in a 10-ml syringe and then over a 70-μm strainer. The remaining tissue pieces were washed with Ca2^+^-Mg2^+^-PBS to remove EDTA, minced thoroughly, and placed into 5 ml HBSS with 10 mM HEPES containing 5% FBS and 0.2 mg/ml collagenase D (Sigma). Tissue was digested at 37°C for 20 min with constant agitation (250 r.p.m.), then vigorously vortexed for 20 s. IELs cell suspensions were collected at 500 g for 10 min at 4°C. Cell pellets were resuspended in 8 ml 44% Percoll (GE healthcare, #57-0891-01) and overlaid over 5 ml of 67% Percoll. Gradients were centrifuged at 500 g for 20 min at 4°C. Thus, cells at the interface were collected and washed with 10 ml PBS. Cells were stained for flow cytometric analysis or used for *in vitro* stimulation.

### Flow Cytometric Analysis

For surface marker analysis, cell suspensions were adjusted to a density of 1 × 106 cells in 50 μl FACS buffer (2% FBS, 0.05% NaN_3_ in PBS). Non-specific binding was blocked by incubation for 10 min at 4°C with anti-CD16-CD32 antibody (BioLegend, clone 93, 5 ng/μl) and stained with the appropriate antibodies for 15 min at 4°C. The following antibodies were used for extracellular staining: CD45 (clone 30F-11, 0.5 ng/μl), CD4 (clone RM4-5, 0.5 ng/μl), TCR-γδ (clone GL3, 4 ng/μl), CD3 (clone 145-2C11, 2 ng/μl), CD25 (clone 3C7, 0.2 ng/μl) from BioLegend. For intracellular staining, cells were first stained for surface markers as detailed above, fixed and permeabilized using Fixation and Permeabilization buffers from eBiosciences following the manufactures' instructions. Briefly, cells were fixed for 30 min at 4°C, washed with Permeabilization Buffer and incubated for 30 min with IL-17A (clone eBio 17B7, 1 ng/μl) or FoxP3 (clone FJK-16 s, 0.5 ng/μl) antibodies in Permeabilization Buffer at 4°C. Cells were washed with FACS buffer, resuspended in 200 μl of FACS buffer and phenotyped on MACSQuant 10 cytometer (Myltenyi Biotec). Analysis of each sample was performed with FlowJo software (version 6, Tree Star).

### Statistics

IBM SPSS 20.0 software was used for statistical analysis. For non-parametric analysis, data were presented as median (interquartile range, IQR) and were analyzed by the Mann-Whitney *U*-test. For the parametric analysis, the unpaired Student's test (two-tailed) was used. For the microbiota analysis, we used the Adonis test implemented in QIIME 1.9.1. Receiver Operator Characteristic (ROC) analysis was performed to assess the performance of the SDI model. Correlations between NIHSS or mRS and the SDI were calculated by the Spearman rank correlation test. Normality of the data was examined using the Shapiro–Wilk test. Subjects were compared using *t*-test, Mann–Whitney test, chi-square, or Fisher's exact test and Wald's test as appropriate. A value of *P* < 0.05 (two-tailed) was considered significant.

Logistic regression analyses were performed for each group to find if the factors were associated to stroke severity or early outcomes. Univariate and multivariate logistic regression analyses were used to assess the association between SDI and stroke severity or early functional outcome. All variables showing a trend in association in univariate analysis (*P* < 0.20) were included (stroke severity model: SDI, WBC, alcohol, serum glucose, HbA1c, Cr and UA; early unfavorable outcome model: SDI, WBC, age, diabetes mellitus, Cr and UA). NIHSS and mRS score were not used in the model since they were part of our definition for stroke severity and outcome. The relative risk was expressed as the odds ratio (OR) with the 95% confidence interval (CI). A value of *p* < 0.05 was considered significant.

## Results

### Model of Stroke Dysbiosis Index (SDI) in Patients With Acute Ischemic Stroke

Patients with large-artery atherosclerotic ischemic stroke according to the TOAST system were recruited for this study. During the study period, a 194-member training group was examined, including 104 patients (72 [69.2%] with anterior ischemic stroke) and 90 healthy individuals. No significant difference was found in age or gender between patients and healthy controls (Table 1).

**Table 1:** Characteristics of the patient-control cohort in the training group.

	**Stroke (*n* = 104)**	**Control (*n* = 90)**	***P*-value**
Males, n (%)	78 (75.0%)	73 (81.1%)	0.307
Age, years	59.38 (12.61)	56.62 (8.16)	0.069
HBP, n (%)	82 (78.8%)	25 (27.8%)	< 0.001
DM, n (%)	46 (44.2%)	1 (1.1%)	< 0.001
CAD, n (%)	4 (3.8%)	0 (0)	0.125
GLU, mmol/L	6.01 (4.06)	4.85 (0.67)	< 0.001
WBC, G/L	7.89 (3.16)	6.60 (2.09)	< 0.001
Cr, μmol/L	77.0 (34.00)	73.5 (17.00)	0.063
UA, μmol/L	357.25 (127.50)	387.5 (110.25)	0.015
TG, mmol/L	1.39 (0.96)	1.40 (1.09)	0.953
TC, mmol/L	4.58 (1.56)	5.055 (1.31)	0.001
HDL, mmol/L	0.91 (0.35)	1.14 (0.38)	< 0.001
LDL, mmol/L	2.98 (1.15)	3.26 (0.94)	0.014
VLDL, mmol/L	0.67 (0.42)	0.67 (0.45)	0.117

*HBP, hyper blood pressure; DM, diabetes mellitus; CAD, cardio artery disease; TC, Total cholesterol; TG, Triglycerides; HDL, high-density lipoprotein; LDL, low-density lipoprotein; VLDL, Very low-density lipoprotein; GLU, glucose; WBC, white blood cell count; Cr, creatinine; UA, Uric Acid. Data were presented as median (interquartile range, IQR) unless otherwise indicated*.

Based on the bacterial 16S rRNA gene data, we identified a total of 18 bacterial genera (FDR_*P* < 0.1, Wilcoxon rank-sum test) that were significantly different between patients and controls, with *Parabacteroides, Oscillospira*, Enterobacteriaceae, etc., enriched in patients, whereas *Prevotella, Roseburia, fecalibacterium*, etc., were enriched in healthy controls (Table 2). A continuous index, the “Stroke Dysbiosis Index” (SDI), was then derived based on the relative abundance of the 18 bacterial genera in the 194-subject training cohort:

(2) $$\begin{array}{l} \text{SDI}=\{\frac{\sum_{i\;=\;7}\text{abundance}\left(\text{stroke enriched}\right)\text{i}}{7}\\ -\;\frac{\sum_{j\;=\;11}\text{abundance}\left(\text{control enriched}\right)\text{j}}{11}\}\times 100 \end{array}$$

**Table 2:** Microbial biomarkers of the stroke (FDR_*P* < 0.1).

**Genera**	**Enriched**	**Genera**	**Enriched**
Knoellia	Control	Butyricimonas	Stroke
Prevotella	Control	Parabacteroides	Stroke
Un Clostridiaceae	Control	Un Rikenellaceae	Stroke
Coprococcus	Control	Un Ruminococcaceae	Stroke
Lachnospira	Control	Oscillospira	Stroke
Roseburia	Control	Bilophila	Stroke
Fecalibacterium	Control	Un Enterobacteriaceae	Stroke
Un Erysipelotrichaceae	Control		
Un Caulobacteraceae	Control		
Un Bradyrhizobiaceae	Control		
Haemophilus	Control		

*FDR_P, False discovery rate of P-values; un, unclassified*.

**Figure 1:**
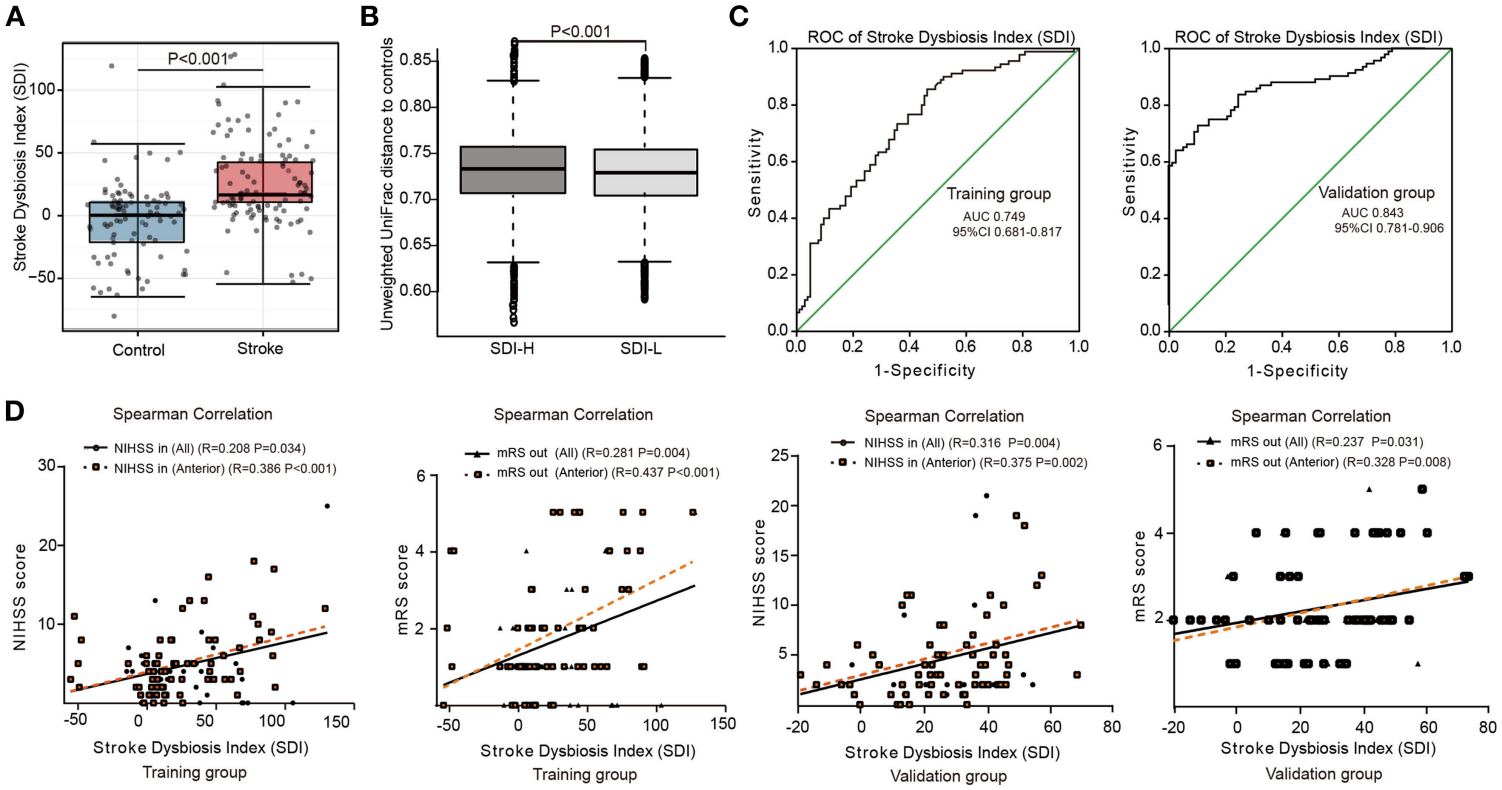
Model of Stroke Dysbiosis Index (SDI) in patients. **(A,B)** The training group. **(A)** Comparison of the SDI between the stroke and control group. **(B)** Distances of higher SDI patient groups (SDI-H) and lower SDI patient groups (SDI-L) to controls. (SDI-H: SDI≥17.0, *n* = 52; SDI-L: SDI < 17.0, *n* = 52) **(C)** Receiver operating characteristic (ROC) curve of the SDI model in the 194-member training group (104 patients and 90 controls) (**C**, left) and the 153-member validation group (83 patients and 70 controls) (**C**, right). **(D)** SDI of stroke patients correlating with the NIHSS score at admission (NIHSS in) and mRS score at discharge (mRS out) in both the training and validation groups. Training group (all patients [*n* = 104]; patients with anterior circulation stroke [*n* = 72]); Validation group (all patients [*n* = 83]; patients with anterior circulation stroke [*n* = 65]). ROC, receiver operating characteristic curve; AUC, area under curve; NIHSS: National Institutes of Health Stroke Scale; mRS: modified Rankin Scale; NIHSS in, recorded on admission; mRS out, recorded at discharge. Spearman's correlation. Boxes denoted the interquartile range (IQR) between the first and third quartiles and the line within denoted the median; whiskers denoted the lowest and highest values within 1.5 times IQR from the first and third quartiles, respectively. Circles denoted data beyond whiskers.

Based on the microbial index model, the median SDI in patients was significantly higher than that in the control group (17.0 vs. 0.45, *P* < 0.05, Wilcoxon rank-sum test) (Figure 1A). Furthermore, we separated the patients into two subgroups: a high-SDI (SDI-H, SDI≥17.0, *n* = 52) group and a low-SDI (SDI-L, SDI <17.0, *n* = 52) group. Higher values of serum glucose, WBC and NIHSS score at admission were observed in SDI-H patients than in SDI-L patients (Table 3). According to the unweighted UniFrac distance analyses, in which the distance represents the dissimilarity between the two microbial communities (19), the distance between the SDI-L group and the control group was significantly shorter than that between the SDI-H group and the control group (*P* < 0.05, Wilcoxon rank-sum test) (Figure 1B), indicating that the gut microbiota of the SDI-L patients was more similar to that of healthy controls than the SDI-H microbiota was.

**Table 3:** Characteristics of patients in the SDI-H and SDI-L groups.

	**SDI-H (SDI ≥ 17, *n* = 52)**	**SDI-L (SDI < 17, *n* = 52)**	***P*-value**
Males, n (%)	38 (73.1%)	40 (76.9%)	0.651
Age, years	64.5 (15.5)	57.0 (17.0)	0.016
HBP, n (%)	43 (82.7%)	39 (75.0%)	0.472
DM, n (%)	30 (57.7%)	16 (30.8%)	0.006
CAD, n (%)	2 (3.8%)	2 (3.8%)	1.000
NIHSS in	5.0 (5.0)	3.0 (3.0)	0.016
NIHSS out	3.5 (5.0)	2.0 (2.0)	0.009
mRS out	2.0 (3.0)	1.0 (1.75)	0.005
Poor Functional outcome (mRS out >2), n (%)	19 (36.5%)	5 (9.6%)	0.001
GLU, mmol/L	6.60 (4.50)	5.28 (2.58)	0.043
WBC, G/L	8.49 (3.47)	7.37 (2.47)	0.010
Cr, μmol/L	85.00 (41.50)	75.00 (30.75)	0.125
UA, μmol/L	358.50 (137.00)	359.25 (123.45)	0.976
TG, mmol/L	1.56 (1.03)	1.35 (1.02)	0.122
TC, mmol/L	4.63 (1.37)	4.56 (1.68)	0.928
HDL, mmol/L	0.91 (0.31)	0.91 (0.35)	0.256
LDL, mmol/L	2.93 (1.19)	3.00 (1.22)	0.743
VLDL, mmol/L	0.67 (0.48)	0.64 (0.43)	0.432

*NIHSS in, recorded at admission; NIHSS/mRS out, recorded at discharge; HBP, hyper blood pressure; DM, diabetes mellitus; CAD, cardio artery disease; TC, Total cholesterol; TG, Triglycerides; HDL, high-density lipoprotein; LDL, low-density lipoprotein; VLDL, Very low-density lipoprotein; GLU, glucose; WBC, white blood cell count; Cr, creatinine; UA, Uric Acid. Data were presented as median (interquartile range, IQR) unless otherwise indicated*.

The discriminative performance of this microbial model at the patient level was assessed using the ROC curve in the stroke and control groups. The area under the ROC curve (AUC) of the SDI model in the training group (the 194-member cohort) was 74.9%. Furthermore, to validate the performance of this microbial model, we included an additional 153 subjects, including 83 patients with acute ischemic stroke (65 [78.3%] with anterior ischemic stroke) and 70 healthy individuals, as the validation group. Analysis of the validation group's intestinal microbiota revealed that the accuracy of diagnosis via the microbial index (SDI model) reached 84.3% in that group (Figure 1C). These results indicate that the SDI index of patients in the acute stage was a reliable indicator of intestinal microbiota dysbiosis in clinical management, with a stable moderate to high patient discrimination performance (AUC 74.9 and 84.3%, respectively) in two independent cohorts.

### SDI Was Positively Related to Patients' Stroke Severity and Poor Early Functional Outcome

To examine the association between microbial dysbiosis and stroke severity, we applied Spearman rank correlation analysis. Interestingly, SDI was positively correlated with the NIHSS score on admission (NIHSS in, *r* = 0.208, *P* = 0.034) and mRS score at discharge (mRS out, *r* = 0.281, *P* = 0.004) in all patients in the training group, and these correlations were especially pronounced in patients with anterior circulation ischemic stroke (NHISS in, *r* = 0.386; mRS out, *r* = 0.437, all *P* < 0.001). Poor functional outcomes (mRS out>2; 34.6% vs. 9.6%, *P* = 0.004) at discharge differed significantly between SDI-H and SDI-L patients (Table 3).

Similar to the training group, SDI in patients in the validation group was positively correlated with the NIHSS score on admission (NIHSS in, *r* = 0.316, *P* = 0.004) and mRS score at discharge (mRS out, *r* = 0.237, *P* = 0.031), especially in those with anterior circulation ischemic stroke (NIHSS in, *r* = 0.375; mRS out, *r* = 0.328; all *P* < 0.05) (Figure 1D).

### SDI Was an Independent Predictor of Stroke Severity and Poor Early Functional Outcome

Next, we used univariate and multivariate analysis to test whether SDI could be used to predict stroke severity and early outcome in the entire patient cohort (i.e., training + validation, *n* = 187). There were 38 (20.32%) patients with severe stroke (NIHSS ≥8). Univariate analyses found that SDI and WBC were associated with severe stroke. DM, alcohol, serum glucose, HbA1c, Cr, and UA showed trends toward association with severe stroke (*P* < 0.2). After adjusting for the above variables in the multivariate regression analysis model, we found that SDI (adjusted OR: 1.019, 95% CI: 1.004–1.034) and WBC (adjusted OR: 1.316, 95% CI: 1.093–1.585) were independent predictors of severe stroke (Table 4).

**Table 4:** Uni-and Multivariable logistic regression analysis for the prediction of stroke severity in the entire patient cohort with acute ischemic stroke.

	***P*-value**	**OR**	**95% CI**
**UNIVARIABLE ANALYSIS**			
Age (years)	0.667	0.994	0.967–1.022
Gender (male)	0.257	1.575	0.718–3.455
SDI	0.001	1.024	1.010–1.038
Hypertension	0.592	1.232	0.575–2.637
Diabetes mellitus	0.175	1.674	0.796–3.520
Cardio artery disease	0.489	0.473	0.057–3.950
Smoke	0.899	0.949	0.421–2.136
Alcohol	0.178	0.405	0.109–1.508
WBC	0.0001	1.322	1.135–1.539
Serum glucose	0.146	1.095	0.969–1.238
Cr	0.076	1.007	0.999–1.015
UA	0.085	0.997	0.993–1.000
TG	0.371	0.790	0.471–1.324
TC	0.889	0.900	0.206–3.929
HDL	1.000	1.000	0.129–7.734
LDL	0.694	1.405	0.258–7.650
HbA1c	0.197	1.131	0.938–1.363
**Model**	***P*****-value**	**Adjust OR** ^**#**^	**95% CI**
**MULTIVARIABLE ANALYSIS**			
WBC	0.004	1.316	1.093–1.585
SDI	0.011	1.019	1.004–1.034
Cr	0.067	1.009	0.999–1.018

*Entire patient cohort, training plus validation cohort (n = 187). Severe stroke, NIHSS score ≥8. SDI, stroke dysbiosis index; TC, Total cholesterol; TG, Triglycerides; HDL, high-density lipoprotein; LDL, low-density lipoprotein; WBC, white blood cell count; Cr, creatinine; UA, Uric Acid; HbA1c, glycosylated hemoglobin*.

# *Adjusted for all above values in table*.

Forty-seven patients (25.13%) had developed unfavorable outcomes, defined by poor functional outcomes (mRS out>2), as of discharge. Univariate analyses found that SDI and WBC were associated with unfavorable outcomes. Age, DM, Cr and UA showed trends toward association with unfavorable outcome (*P* < 0.2). In the logistic regression model, we found that only SDI (adjusted OR: 1.022, 95% CI: 1.008–1.035) was an independent predictor of early unfavorable outcome (Table 5).

**Table 5:** Uni-and Multivariable logistic regression analysis for the prediction of early outcome in the entire patient cohort with acute ischemic stroke.

	***P*-value**	**OR**	**95% CI**
**UNIVARIABLE ANALYSIS**			
Age (years)	0.139	1.023	0.992–1.055
Gender (male)	0.969	0.984	0.429–2.258
SDI	0.0001	1.024	1.011–1.037
Hypertension	0.757	0.880	0.390–1.982
Diabetes mellitus	0.147	1.698	0.831–3.470
Cardio artery disease	0.745	0.764	0.151–3.875
Smoke	0.845	1.090	0.483–2.463
Alcohol	0.606	0.757	0.263–2.179
WBC	0.007	1.209	1.052–1.389
Serum glucose	0.215	1.124	0.934–1.353
Cr	0.091	1.010	0.999–1.021
UA	0.071	0.998	0.995–1.001
TG	0.907	1.025	0.679–1.547
TC	0.919	1.073	0.276–4.176
HDL	0.399	0.440	0.065–2.961
LDL	0.833	0.845	0.177–4.032
HbA1c	0.961	0.993	0.754–1.308
**Model**	***P*****-value**	**Adjust OR** ^**#**^	**95% CI**
**MULTIVARIABLE ANALYSIS**			
SDI	0.001	1.022	1.008–1.035
WBC	0.055	1.158	0.997–1.344

*Poor early outcome, mRS score >2*.

*^#^Adjusted for all above values in table*.

### Transplantation of the SDI-H Patient Microbiota Exacerbated the Brain Injury in HFA Mice

**Figure 2:**
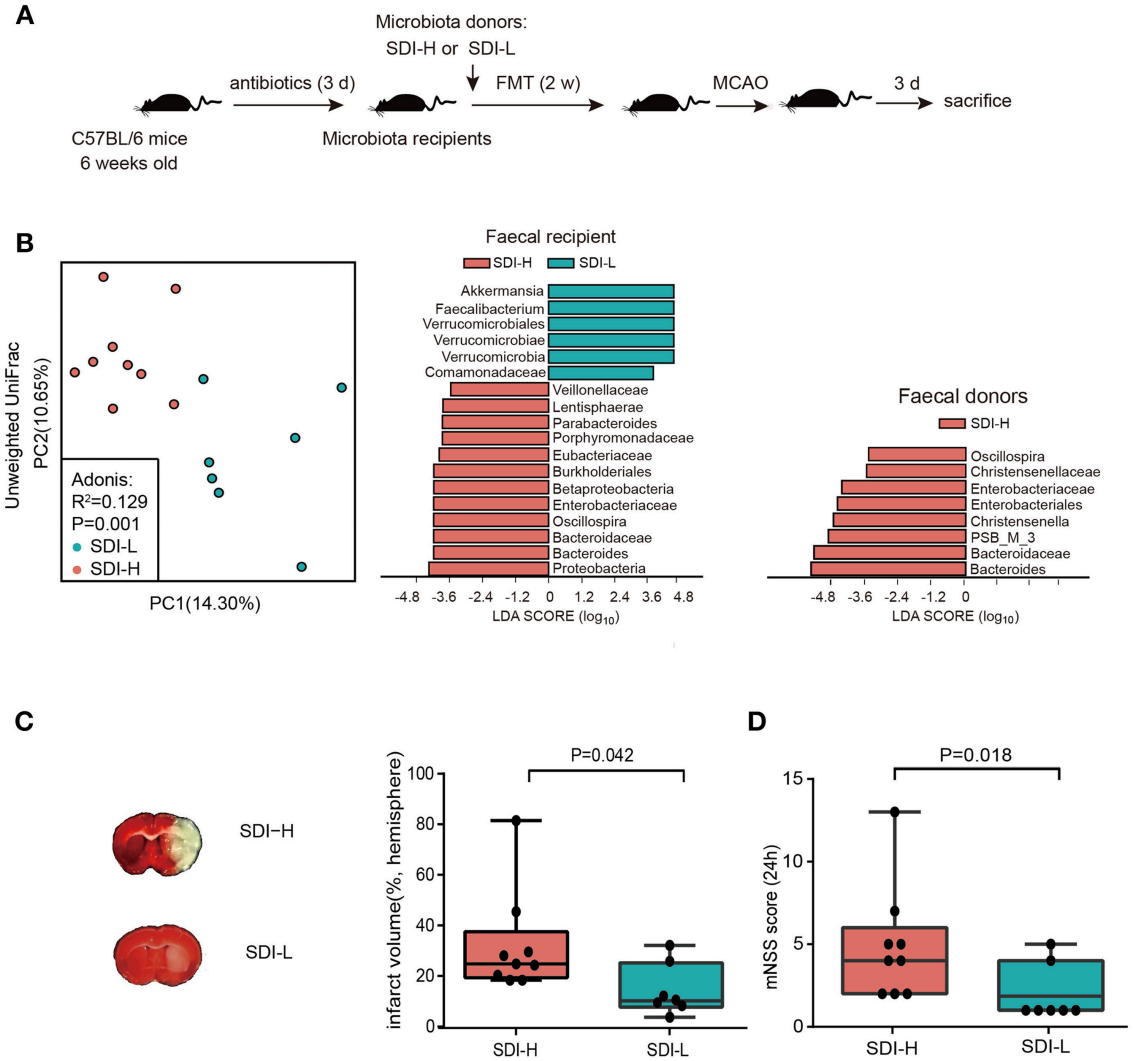
Causality experiment in mice model. **(A)** Animal experiment. **(B)** The principle coordinate analysis (PCoA) result showed the group patterns of the SDI-H and SDI-L recipient mice group based on the unweighted UniFrac distances (SDI-H, *n* = 9; SDI-L, *n* = 7) (**B**, left). The Linear discriminant analysis effect size (LEfSe) identified the most differentially abundant taxon between two recipient mice groups (**B**, middle) or two donor groups (**B**, right). Four genera (i.e., *Oscillospira*, Enterobacteriaceae, *Bacteroides*, and Bacteroidaceae) enriched in SDI-H donor feces (**B**, right) were successfully transplanted to SDI-H recipient mice (**B**, middle). **(C)** Representative Images of TTC-stained ischemic lesion (**C**, left) and quantification of infarct volume ratio accounting for hemisphere (**C**, right) in SDI-H and SDI-L mice 3 d after MCAO induction. **(D)** Modified neurological severity scores at day 1 (24 h) after MCAO. FMT indicated fecal microbiota transplantation. MCAO, middle cerebrovascular artery occlusion; PC, principle coordinate analysis (PCoA). Boxes denoted the interquartile range(IQR) between the first and third quartiles and the line within denoted the median; whiskers denoted the lowest and highest values within 1.5 times IQR from the first and third quartiles, respectively. Circles denoted data beyond whiskers.

Furthermore, animal experiments were conducted to investigate the potential causal effect of patients' disturbed microbes on brain injury. HFA mice were established via FMT into antibiotic-induced abiotic mice. In order to improve the stability of microbial colonization, we repeated FMT in recipient mice by oral gavage every day for 2 weeks (Figure 2A). After 2 weeks of FMT treatment, the abiotic mice that received SDI-H patients' fecal microbiota showed significant differences in gut microbiota compared to SDI-L recipient mice upon PCoA analysis (*R*^2^ = 0.129, *P* = 0.014, Adonis test) (Figure 2B, left). The results showed that four genera (i.e., *Oscillospira*, Enterobacteriaceae, *Bacteroides*, and Bacteroidaceae) enriched in SDI-H donor feces (Figure 2B, right) were successfully transplanted to SDI-H recipient mice (Figure 2B, middle). We further assessed the SDI in mice, and the results showed that SDI-H recipient mice had a significantly higher SDI than SDI-L recipient mice (median, 9.271 vs. 7.052, *P* = 0.008, Mann-Whitney test) (Supplementary Figure 1A). These results confirmed the success of FMT treatment.

Both groups of mice were subjected to MCAO surgery after the 2-week FMT treatment. The overall mortality rate was 10% in both the SDI-L and SDI-H groups. Two mice in the SDI-L group lacked brain ischaemia as quantified postmortem by histological analysis and were therefore excluded. No significant difference in mortality was found between these two groups (*P* = 1.000, Fisher's exact test). Remarkably, SDI-H mice had increased infarct volumes after ischemic lesions (Figure 2C) in the MCAO stroke model and exacerbated neurological functional impairment (Figure 2D), with increased pro-inflammatory (IL-17^+^) γδ T cells and reduced (CD4^+^CD25^+^) T cells in the spleen (Supplementary Figures 1B,C).

**Figure 3:**
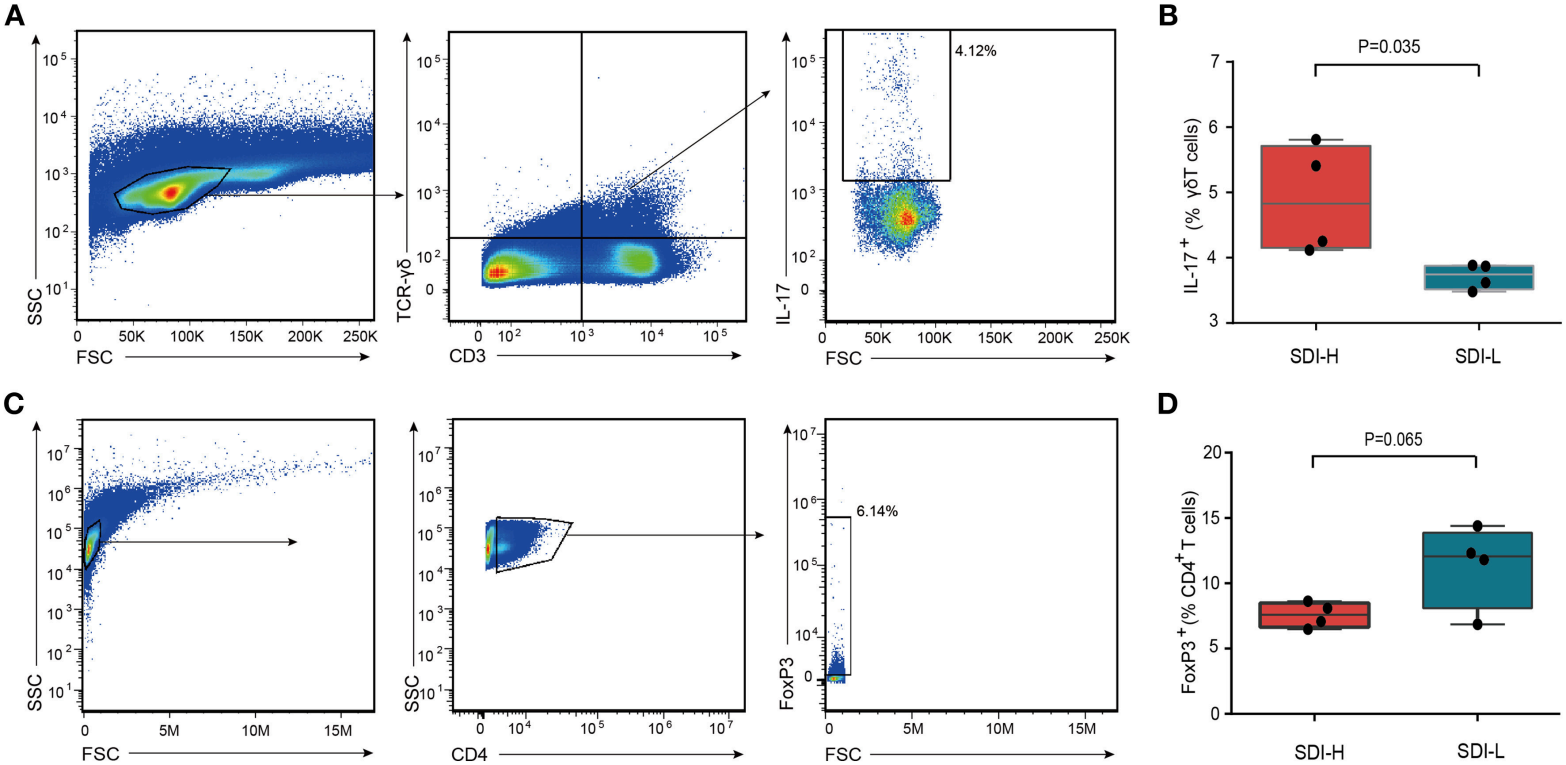
Increased IL-17^+^ γδ T cells in the small intestine of SDI-H recipient mice. **(A)** Flow cytometry analysis of IL-17 production in γδ T cells (CD3^+^ TCR-γδ^+^) in the iELs (intestinal intraepithelial lymphocyte) of the small intestine from SDI-H and SDI-L mice 3 days after MCAO surgery. The boxes in the center identify IL-17^+^ cells and numbers represent IL-17^+^ cells as a percentage of γδ T cells in SDI-H and SDI-L mice. **(B)** The bar graphs indicate percentage of IL-17-producing cells in iELs of the small intestine (SDI-H, *n* = 4 and SDI-L, *n* = 4). **(C)** Representative flow cytometry plots. CD4^+^ T cells were identified in side scatter (SSC)/ forward scatter (FSC) plots and Treg cells (CD4^+^ Foxp3^+^) in the iELs of the small intestine of SDI-H mice and SDI-L mice. **(D)** Graphs represent percentages of FoxP3^+^ cells in the iELs of the small intestine (*n* = 4 per group). Columns represent mean ± s.e.m. Students' *t*-test.

We further assessed the γδ T and Treg cells in the small intestine of SDI-H and SDI-L mice (Figures 3A,C). The results showed that SDI-H mice had significantly more (IL-17^+^) γδ T cells (Figure 3B) and a trend toward fewer Treg (CD4^+^Foxp3^+^) cells (Figure 3D) among the intestinal intraepithelial lymphocytes (iELs) of the small intestine than SDI-L mice had; these differences are suggested to be associated with the increased infarct volume in brain (11, 13).

## Discussion

In current clinical practice, the gut microbiota value is usually examined only in retrospect in the context of stroke because of the complex changes in microbial taxa and the heterogeneity of the stroke population. Our previous study has demonstrated the significantly complex changes in gut microbiota (dysbiosis) of stroke patients compared to healthy controls; but no further interpretation of the relationship between gut microbiota dysbiosis and stroke was demonstrated (3). On the basis of our previous study, this study focuses on establishing an index model for measuring the gut microbiota dysbiosis in stroke patients, which may contribute to a better clinical application of gut microbiota data in stroke. The SDI, a gut microbiota index representing the patterns of gut microbiota dysbiosis, in stroke patients, was established and validated in this study. Among patients, higher values of this microbial index SDI was closely related to more severe brain injury and an increased probability of unfavorable outcomes. A logistic regression model showed that SDI was an independent predictor of severe stroke and early unfavorable outcome in patients. Fecal microbiota in patients with higher SDI aggravated acute brain injury in a mouse model, indicating the harmful effects of patients' disturbed gut microbes on stroke prognosis, which may propose new intervention targets for stroke patients.

In this study, SDI, a novel model established for assessing the gut microbiota data in stroke, revealed the presence of bacterial dysbiosis in patients with acute ischemic stroke. Patients with higher SDI had increased abundance of Enterobacteriaceae and *Parabacteroides* and decreased abundance of *fecalibacterium*, Clostridiaceae, and *Lachnospira*. In IBD patients, increased Enterobacteriaceae and decreased Clostridiales (containing Clostridiaceae) are strongly correlated with IBD severity and treatment effectiveness (6). At the beginning of the pre-transplant conditioning regimen, high Enterobacteriaceae was a marker for the risk of microbiologically confirmed sepsis, and lower Lachnospiraceae (containing *Lachnospira*) was the only factor for increased risk of overall mortality (24). Thus, the enriched intestinal Enterobacteriaceae and reduced Clostridiaceae and *Lachnospira* in SDI-H patients may be related to the elevated inflammatory response or infections post stroke, which are associated with severe brain injury and poor stroke outcome. *Fecalibacterium* is considered to have anti-inflammatory properties and contribute to gut health through butyrate production (25), which can increase Foxp-3 and IL-10 expression by favoring Treg differentiation. In this study, mice that received SDI-H patients' microbiota had increased Enterobacteriaceae and decreased *fecalibacterium* in the gut microbiota and developed exacerbated brain injury with a trend toward decreased neuroprotective Treg differentiation. Thus, a significant reduction of *fecalibacterium* in SDI-H patients could potentially lead to severe brain injury with the modulation of T cells.

The SDI model in the present study was not simply established for stroke identification. Indeed, it is known that the abrupt onset of focal neurological deficits is the hallmark of the diagnosis of ischemic stroke, and current methods, especially neuroimaging (i.e., computed tomography imaging [CT] and magnetic resonance imaging [MRI]), are the most readily available method for stroke identification with highly accurate performance. With a stable moderate to high patient discrimination performance (AUC 74.9 and 84.3%, respectively) in two independent cohorts, the SDI index of patients in the acute stage was a reliable indicator of intestinal microbiota dysbiosis in clinical management. Data from our recent study showed that the disturbed gut flora were relatively stable during hospitalization. Changes in gut microbiota were observed a few days (1–4) after stroke and persisted with little change for at least 3 weeks (26). Similarly, a previous study reported that the gut microbiome was relatively stable after stroke (4). Based on this relatively reliable microbial-index model, we also found the significantly positive correlations between SDI and stroke severity and early functional outcome in these two patient cohorts, which provide additional convincing evidence.

Outcome assessments at discharge, such as mRS score, are often used in stroke researches (27, 28). In the present study, we used the mRS scores at discharge for outcome assessment; and the median of length of hospital stay of these patients was 7–10 days. Early recovery trajectory, such as 7-day follow-up, captures the largest proportional recovery per unit time and strongly determines eventual outcome (29, 30). Patients after day 7 of onset usually attain a relatively stable state (31) and almost half of them are discharged from the hospital around this time (32). Although the 90-day mRS score is an important outcome assessment in stroke, which was not assessed in the present study. However, the early outcome assessment is also important and useful for doctors to adjust treatment plans or to develop novel treatment approaches. Interestingly, our study for the first time showed that gut microbiota dysbiosis, indicated by SDI, has a potential to be an independent predictor of early outcome in patients with acute ischemic stroke. Identifying risk factors for poor stroke outcome is important for clinical management. To assess early improvement or final functional outcome, parameters including age (33, 34), diabetes (33, 35), hypertension (34), brain lesion site (36, 37), UA (35), and serum glucose (35) have been tested. However, inconsistent results were reported with many of these parameters (33–37), and their correlation with early outcome was less obvious in the present study.

In this paper, the multivariate logistic analysis found that SDI (OR: 1.022, 95% CI: 1.008–1.035) was an independent predictor of early unfavorable outcome (defined as an mRS score >2), while other clinical parameters (including age, gender, hypertension, DM, cardiac artery disease, smoking, alcohol, serum glucose, Cr, UA, TG, total cholesterol [TC], high-density lipoprotein [HDL], low-density lipoprotein [LDL], and HbA1c) had no significant correlation with poor early outcome. Concerning the limitation of the relatively small patient number in this study, larger multicenter cohorts were needed to validate the predictive performance of SDI for stroke outcome. However, these founding highlight gut microbiota dysbiosis as a potential risk factor of poor outcome in stroke patients, shedding new light on the relationship between microbiota and human diseases. In addition, advances in DNA sequencing technologies and data analysis have provided drastic improvement in microbiome analyses (38). Detection and analysis of gut microbiota is maturing with less labor and time than ever before. It takes < 1 day to complete the whole transaction of gut microbiota analysis (including DNA extraction, PCR sequencing amplification, data analysis) in our experiment. The future potential for the gut microbiota examination in improving the prediction of early functional outcome, as well as advancing current clinical practice, is promising.

Human flora-associated (HFA) animals were used in this study to determine whether there was a causal relationship between gut dysbiosis and stroke outcome. Compared to mice which received a fecal microbiota transplantation of feces from patients of low SDI, mice transplanted with high-SDI patients' microbiota developed worse stroke outcomes, with larger infarct volume in the brain and an increased proportion of IL-17^+^ γδ T cells of the small intestine compared to the SDI-L group. These results indirectly confirm the potential causal effect of patients' disturbed gut microbiota on poor stroke outcome and provide new targets for clinical treatment. Within animals, a previous study has demonstrated the correlation between the gut microbiota dysbiosis and the severity of brain injury via two distinct stroke models (13). As described in the previous research (13), severe stroke (induced by the filament middle cerebral artery occlusion [fMCAO] model) caused the significant gut microbiota dysbiosis in mice, while mild stroke (induced by the distal middle cerebral artery occlusion [dMCAO] model) caused little change in gut microbiota. In turn, post-stroke dysbiosis caused by severe stroke exacerbated lesion volume and functional deficits after experimental stroke. Normalization of brain lesion-induced dysbiosis via therapeutic transplantation of fecal microbiota improved stroke outcome. These results supported the correlation between gut microbiota dysbiosis and stroke severity within mice, in which gut microbiota dysbiosis and stroke severity were influenced and interacted as both cause and effect. Although the role of gut microbiota on brain injury was demonstrated in recent animal studies (11, 13), the harmful effect of patients' disturbed gut microbiota on brain injury via HFA mice model in this study may provide much direct information on the precise role of patients' gut microbiota on stroke outcome. Nevertheless, the causal effect of gut microbiota on stroke outcome was preliminarily explored in this study. Further investigations are required to explore the reasons for the difference of SDI in gut microbiota among patients, as well as the detailed mechanisms through which gut microbiota affect stroke in patients.

In microbial transplantation studies, germ-free (GF) mice were the first choice and best controlled model in long-term microbiota transplantation (39). However, the difficulties of generating the GF mice of interest and restrict conditions for maintaining their health status are challenges in the use of GF mice. Depletion of the gut microbiota by antibiotic use is an alternative to GF technology; but there are some problems to be considered when using the antibiotic mouse model for the fecal transplantation experiments: (1) Bacteria left: Although a broad-spectrum antibiotic kills a majority of bacterial species, there are still some bacteria left in the gut that may have an impact on colonization and stability over time. It is impossible to control the exact effect of antibiotic treatment in terms of which species are fully eradicated and which are only reduced. (2) The effects of gut microbiota on the immune system should be taken into consideration. Exposure to microbes before depletion with antibiotics can have long-lasting effects on host physiology as the immune system is known to be primed by the gut microbiota in early life (40, 41). The immune system in GF mice has never encountered bacteria, and that of antibiotic-treated mice, in contrast, has been stimulated with prior commensal bacteria. This difference should be considered, at least for certain disease models, which may yield different outcomes after the successful colonization. (3) Antibiotic treatment can result in an overgrowth of a few species, such as *Klebsiella* spp., which may detrimental to the health of the animal or have a substantial dominant role in the microbial profile (42). (4) Direct effects of antibiotics on host physiology. The direct effects of antibiotics on the host were also recently systematically investigated, with repression of mitochondrial and ribosomal function as remarkable findings in GF mice treated with antibiotics (43). (5) Moreover, the risk of favoring bacteria with antibiotic resistance genes should be considered in non-therapeutic use (44). Nevertheless, the host phenotypes also can be transferred via fecal transplantation to antibiotic-treated mice, which was less labor-demanding and more cost efficient compared to GF mice. Thus, the antibiotic-treated mice were commonly utilized in fecal microbial transplantation experiments (39).

The gut microbiota appears to be a potential target for diseases that constitute risk factors for stroke and for complications after stroke. Compared with the SDI-L patients, the SDI-H patients were older and had a higher proportion of diabetes, both of which are associated with gut microbiota (45, 46). Aging affects most of the multi-cellular organisms, constraining longevity and lead to neurodegenerative diseases including stroke. Probiotics, or preferably a combination of probiotics and prebiotics, can modify the composition of the gut microbiome and reduce the impact of aging and the risk of neurodegenerative diseases (47, 48). Tree nuts, as a good source of prebiotics, have been suggested to exert anti-aging effect (49). Several probiotics have been shown to benefit the human immune system (50). Prebiotics or probiotics may be a good treatment for SDI-H patients. However, little research has been performed on treatments targeting microbiota with prebiotics or probiotics in clinical stroke experiments. Further evidence is needed to confirm the effects of prebiotics or probiotics on stroke outcome.

Colonization with normal bacteria may be a potential approach to modify the disturbed gut microbiota in stroke patients, especially those with higher SDI value. Transfer of a whole microbiota community from a healthy donor, so-called FMT has been established in the treatment of patients with severe *C. difficile* colitis; and FMT has been used as a relatively safe and successful medical therapy treatment in patients with active IBD (51, 52). Normalization of brain lesion-induced dysbiosis via therapeutic transplantation of fecal microbiota improved stroke outcome in animal experiments (13). In current clinical practice, stroke patients are often treated with antibiotics due to post-stroke infections. However, not only pathogens in the target organ (i.e., lung or urinary tract) but also the commensal bacterial populations are systematically be influenced by antibiotics treatment. Antibiotics treatment might shift the gut microbiome and lead to detrimental outcome of stroke. In animal experiments, extensive microbiota depletion with broad-spectrum antibiotic pretreatment worsened the outcome of stroke due to acute and severe colitis (10). In contrast, colonization with normal microbiota will improve the post-stroke outcome (10). The above results highlight that restoring a balanced community may contribute to the personalized therapy for optimal outcomes after ischemic stroke. However, the field of microbial therapy is in its infancy and little is known about side effects.

The present study has several limitations. First, the association between SDI and long-term functional outcome in these patients was unclear, which needed follow-up clinical studies to elucidate. Second, the underlying mechanism through which gut dysbiosis in patients affects brain injury in patients is still unclear. Third, the mouse experiment was not very extensive. Additional studies are needed to explore the detailed mechanisms by which gut microbiota affect stroke in patients. Furthermore, additional multicenter studies with larger sample sizes (including other stroke subtypes) are needed to validate the universality and reliability of this microbial index model.

Despite these limitations, this study offers a novel model scheme to address gut microbiota dysbiosis in patients with acute ischemic stroke, which may provide a basis for future study of clinical applications of gut microbiota in stroke. SDI model revealed that the composition of the gut microbiota was associated with brain injury in patients and could be a novel potential predictor of stroke severity and early outcome in stroke. Our study identified gut microbiota dysbiosis as a potential risk factor for unfavorable stroke outcome, and the gut microbiota was revealed as a potential therapeutic target for stroke.

## Ethics Statement

The study was conducted in accordance with the principles of the Declaration of Helsinki and received approval from the Ethical Committee of Southern Medical University. Written informed consent was obtained from all study subjects or their legal guardians by neurologists prior to data and sample collection. Animals used in this experiment were treated in accordance with the National Institutes of Health guide for the care and use of Laboratory animals (NIH Publications No. 8023, revised 1978). The protocols were approved by the Animal Ethics Committee of Nanfang hospital, Southern Medical University.

## Author Contributions

JY, YH, and H-WZ conceived and designed the experiments. G-HX, CY, X-LZ, X-XG, K-YX, R-TX, J-JZ, Q-HW, and C-HT performed the experiments. G-HX, CY, X-LZ, H-WZ, and YH analyzed the data. G-HX wrote the paper. All authors are responsible for the writing, reviewing, and editing of this manuscript.

### Conflict of Interest Statement

The authors declare that the research was conducted in the absence of any commercial or financial relationships that could be construed as a potential conflict of interest.
