# Supplementary figures and images for: Stroke Dysbiosis Index (SDI) in Gut Microbiome Are Associated With Brain Injury and Prognosis of Stroke
Source: Front Neurol. 2019 Apr 24;10:397. doi: 10.3389/fneur.2019.00397 (PMC6491752; doi:10.3389/fneur.2019.00397)

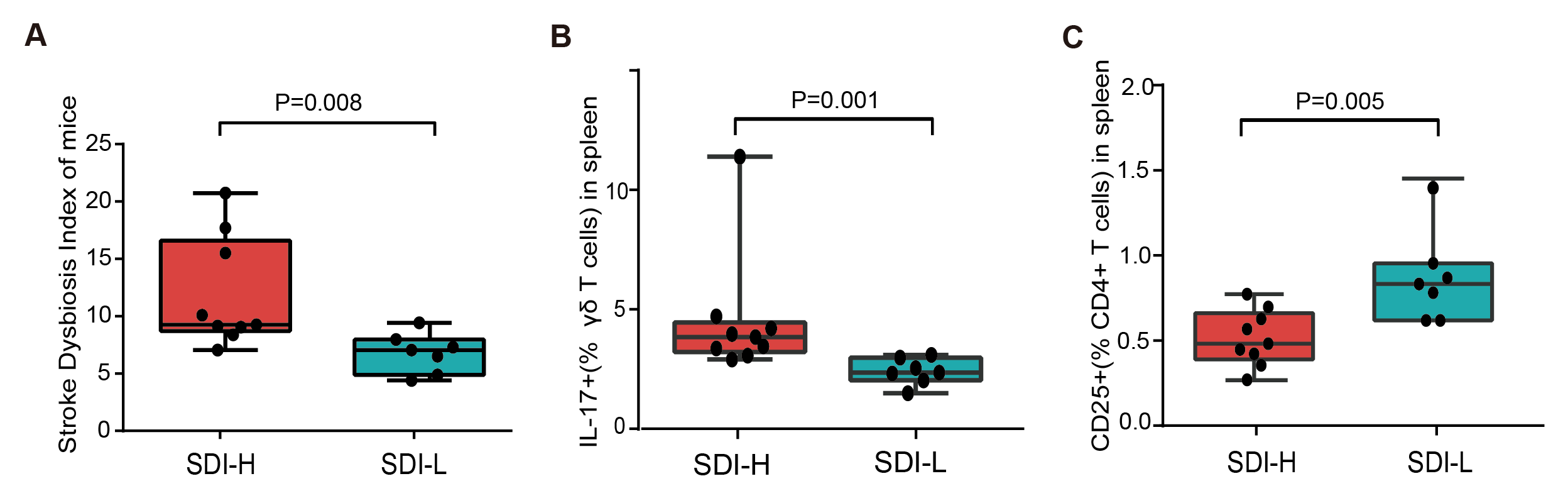

Supplement: Supplementary file 1 [file Image_1.TIF]
